# Diagnostic Yield and Clinical Utility of Chromosomal Microarray Analysis (CMA) in Children With Developmental Delay, Intellectual Disability, and Autism Spectrum Disorder: A Systematic Review

**DOI:** 10.7759/cureus.115483

**Published:** 2026-08-31

**Authors:** Muna Omer Abdelgayoum Ahmed, Einas Abdelmoneim Khiri Ahmed, Sarra Elnour Ahmed Elnour, Arafa MohamedAhmed Mohamed Elamin, Hagar Elhag Mohamed Elamin, Sahar Ali Osman Mohamed Elawad, Nermin Ezat Elbakry Abdelmahdy, Aliaa Salah Alquhazi

**Affiliations:** 1 Pediatrics, University Hospital Kerry, Kerry, IRL; 2 Pediatrics, Al Quwayiyah Hospital, Al Quwayiyah, SAU; 3 Pediatrics Emergency, Armed Forces Hospital Southern Region, Khamis Mushait, SAU; 4 Pediatrics, Maternity and Children's Hospital, Tabuk, SAU; 5 Pediatrics, Shaqra General Hospital, Shaqra, SAU; 6 Pediatrics, Armed Forces Hospital Jazan, Jazan, SAU; 7 Pediatrics, Ashford and St. Peter's Hospital, Chertsey, GBR; 8 Pediatrics, East Kent Hospitals University NHS Foundation Trust, Kent, GBR

**Keywords:** autism spectrum disorder, chromosomal microarray, copy number variation, developmental delay, diagnostic yield, intellectual disability, systematic review

## Abstract

Children with unexplained developmental delay (DD), intellectual disability (ID), and autism spectrum disorder (ASD) frequently undergo chromosomal microarray analysis (CMA). Two distinct questions arise: how often CMA identifies a causative variant, and whether that result changes clinical care. The purpose of this systematic review is to synthesize contemporary primary evidence on (i) the diagnostic yield of CMA in children with DD, ID, and/or ASD and the determinants of that yield, and (ii) the downstream clinical utility of a CMA result. Four electronic databases were searched from 1 January 2021 to 26 June 2026, with no language or country restriction. Eligible studies were original primary cohort or case-series analyses reporting patient-level diagnostic yield of postnatal CMA in pediatric DD/ID/ASD populations; non-original publication types were excluded. Methodological quality was appraised using a validated critical appraisal checklist for case series with pre-specified decision rules, and findings were synthesized narratively. Of 434 records identified, 13 studies met the eligibility criteria, comprising 11,535 children from nine countries. Study-level yield of pathogenic and likely pathogenic copy number variants ranged from 11.0% (95% CI 9.4-12.9) to 37.2% (95% CI 27.3-48.3); the median study-level yield was 18.5%, and 11 of 13 cohorts fell between 14% and 23%. Yield was lower in ASD (3.3-15.2%) than in DD/ID phenotypes (17.1-33.6%) and reached 27.4% where DD/ID co-occurred with multiple congenital anomalies. Variants of uncertain significance affected 2.7-12% of children. In the single cohort testing both modalities in the same patients, CMA yield exceeded karyotype yield (16.0% vs 7.0%). Seven studies were at low risk of bias, two at moderate risk of bias, and four at high risk of bias. No included study prospectively documented a change in clinical management. Study-level CMA yields were broadly consistent across diverse health systems, with a median of 18.5%. Yield is strongly phenotype-dependent, supporting pre-test phenotypic stratification rather than undifferentiated testing. Because no included cohort measured downstream management change, the clinical utility of CMA could not be quantified from contemporary evidence and remains a priority for prospective study.

## Introduction and background

Global developmental delay (GDD), intellectual disability (ID), and autism spectrum disorder (ASD) together constitute the most prevalent group of chronic pediatric neurodevelopmental conditions, and a substantial proportion of affected children never receive an etiological diagnosis despite prolonged investigation. Genetic factors are thought to underlie approximately half of all cases of unexplained DD/ID, with structural genomic variation representing one of the largest identifiable contributors [1]. Population surveillance data from the United States indicate that developmental disabilities affect a rising proportion of children, generating a correspondingly expanding demand for etiological evaluation [2]. Establishing a molecular diagnosis is not merely an academic exercise: it terminates the diagnostic odyssey, permits condition-specific surveillance, informs prognosis and recurrence-risk counseling, and enables families to access syndrome-specific support networks and clinical trials [3].

Because the tests discussed in this review are often unfamiliar outside genetics, it is worth stating in plain terms what each one does. A conventional karyotype photographs the 46 chromosomes under a microscope and detects only large rearrangements, roughly five to ten million base pairs or larger. Chromosomal microarray analysis (CMA) instead compares the amount of DNA present at many thousands of positions across the genome against a reference and so detects missing (deleted) or extra (duplicated) stretches of DNA--collectively copy number variants (CNVs)--that are far too small to see down a microscope, typically down to tens of thousands of base pairs. Two array formats are in common use: array comparative genomic hybridization (aCGH), which compares patient and reference DNA directly, and single-nucleotide polymorphism (SNP) arrays, which additionally read individual DNA letters and can therefore detect long runs of identical DNA inherited from both parents, revealing consanguinity or uniparental disomy (both copies of a chromosome inherited from one parent). What CMA cannot do is read the DNA sequence itself, so it is blind to single-letter spelling changes within a gene; detecting those requires exome sequencing (ES), which reads the protein-coding portion of all genes, or genome sequencing (GS), which reads the whole genome essentially.

On the basis of a large evidence synthesis, an international consensus statement in 2010 designated CMA the first-tier clinical diagnostic test for individuals with DD/ID, ASD, or multiple congenital anomalies, reporting a diagnostic yield of approximately 15-20% compared with roughly 3% for karyotype [4]. Interpretation of the resulting CNVs has since been progressively standardized, most notably through the joint technical standards issued by the American College of Medical Genetics and Genomics (ACMG) and the Clinical Genome Resource (ClinGen), which introduced an evidence-weighted scoring framework intended to improve classification concordance between laboratories [5]. Nonetheless, interpretation remains difficult for a substantial subset of findings. Many recurrent neurodevelopmental CNVs, including those at 15q11.2, 15q13.3, and 16p11.2, show incomplete penetrance and variable expressivity, are inherited from mildly affected or unaffected parents, and may require a second genomic or genic "hit" to produce a clinical phenotype, complicating both classification and counseling [6].

The diagnostic landscape has also shifted since the original first-tier recommendation. A multidisciplinary meta-analysis and consensus statement concluded that exome sequencing achieves a higher molecular diagnostic yield than CMA in neurodevelopmental disorders and argued for its adoption as a first-tier test [7]. This position was subsequently formalised in the first evidence-based ACMG clinical guideline on the subject, which used a Grading of Recommendations Assessment, Development and Evaluation (GRADE) evidence-to-decision framework and strongly recommended that ES or GS be considered as a first- or second-tier test for paediatric patients with congenital anomalies, developmental delay or intellectual disability, on the grounds of higher diagnostic yield, demonstrable effects on clinical management and reproductive planning, and relatively few harms [8]. More recently, a synthesis of 102 studies comprising 55,752 children reported a pooled diagnostic yield of 0.37 for exome sequencing against 0.19 for CMA in global developmental delay and intellectual disability [9]. Yet in many health systems, including much of South Asia, South-Eastern Europe, Latin America, and the Middle East, sequencing-based testing remains unavailable, unreimbursed, or restricted to specialist centers, and CMA continues to function as the practical entry point to genomic diagnosis.

Against this background, contemporary primary data on how CMA actually performs across heterogeneous real-world pediatric populations remain directly relevant to clinical decision-making. This systematic review addresses two questions that are frequently conflated and are kept deliberately separate throughout: first, what is the diagnostic yield of CMA in children with DD, ID, and/or ASD, and which phenotypic, demographic and technical features predict it; and second, what is the downstream clinical utility of a CMA result, meaning any documented change in surveillance, treatment, referral or reproductive counseling. A third question--the formal diagnostic accuracy of CMA, expressed as sensitivity, specificity, predictive values, discrimination, and calibration--is addressed only to establish that it cannot be answered from this literature, and the reasons are set out explicitly. As will be shown, the contemporary literature answers the first question consistently, the second scarcely at all, and the third not at all--and that asymmetry, rather than the headline yield figure, is the principal message of this review.

## Review

Materials and methods

Protocol and Reporting

This systematic review was designed, conducted, and reported in accordance with the Preferred Reporting Items for Systematic Reviews and Meta-Analyses (PRISMA) 2020 statement [10]. The review was not prospectively registered in PROSPERO or any equivalent public registry. A written internal protocol specifying the review questions, Population, Intervention/Exposure, Comparator, Outcomes and Study design (PICOS) eligibility criteria, outcome definitions, the five-year date restriction and the planned narrative synthesis was finalized and dated by the review team before screening commenced, and no amendments were made to it thereafter; because this protocol is not publicly deposited, however, readers cannot independently verify that these decisions were pre-specified, and this is stated again among the limitations.

Eligibility Criteria

Eligibility was defined a priori using the Population, Intervention/Exposure, Comparator, Outcomes and Study design (PICOS) framework, a structured tool for specifying review eligibility criteria and guiding search construction [11] (Table 1). Eligible studies were original, peer-reviewed primary research articles reporting the patient-level diagnostic yield of postnatal CMA in pediatric or predominantly pediatric cohorts ascertained for DD, global developmental delay (GDD), ID, and/or ASD. The distinction between patient-level and variant-level yield was decisive for eligibility and is worth stating explicitly: patient-level yield takes the number of children tested as its denominator and answers the clinical question "what proportion of children investigated received a diagnosis?", whereas variant-level yield takes the number of CNVs detected as its denominator and answers the laboratory question "what proportion of detected variants were pathogenic?". A single child may carry several CNVs, and reports giving only the latter do not permit the number of diagnosed children to be recovered; such reports were therefore excluded. Narrative and systematic reviews, meta-analyses, conference abstracts, editorials, commentaries, letters, protocols, preprints, case reports, and series of fewer than 20 participants were excluded, as were prenatal studies and adult-only cohorts. No geographical, national, or language restriction was applied at any stage: studies from any country and in any language were eligible.

**Table 1:** Eligibility criteria based on the PICOS framework. ASD, autism spectrum disorder; aCGH, array comparative genomic hybridization; CMA, chromosomal microarray analysis; CNV, copy number variant; DD, developmental delay; GDD, global developmental delay; ID, intellectual disability; POC, products of conception; SNP, single-nucleotide polymorphism; UPD, uniparental disomy; VUS, variant of uncertain significance; FISH, fluorescence in situ hybridization; MLPA, multiplex ligation-dependent probe amplification; PICOS, Population, Intervention/Exposure, Comparator, Outcomes and Study design.

Domain	Included	Excluded
Population	Children/adolescents (0–18 y, or predominantly pediatric cohorts) clinically ascertained for unexplained DD, GDD, ID, and/or ASD, with or without dysmorphism, congenital anomalies or epilepsy	Adult-only cohorts; prenatal or POC specimens; cohorts without a neurodevelopmental indication; pre-existing confirmed molecular diagnosis
Intervention/exposure	Postnatal CMA (aCGH, SNP array or combined platforms) on blood or saliva DNA, performed diagnostically	Karyotype, FISH, MLPA, gene panels, exome, or genome sequencing as the sole index test; prenatal CMA
Comparator	None required (single-arm yield acceptable). Karyotype, prior targeted testing or sequencing on the same cohort extracted where reported	Absence of a comparator was not itself an exclusion
Outcomes	Primary: patient-level diagnostic yield, i.e., the proportion of tested children in whom ≥1 pathogenic/likely pathogenic CNV or pathogenic UPD was found (denominator = children tested). Secondary: VUS rate; phenotype-stratified yield; CNV type, size, and inheritance; comparative karyotype yield; reported clinical utility	Studies reporting only variant-level yield, i.e., counts or proportions of CNVs classified as pathogenic among all CNVs detected (denominator = variants, not children), from which the number of diagnosed children cannot be recovered; novel-locus discovery without cohort-wide yield
Study design	Original peer-reviewed primary research: prospective/retrospective cohorts, consecutive or clinic-based case series (n ≥ 20), cross-sectional diagnostic studies	Reviews, systematic reviews, meta-analyses; conference abstracts; editorials, commentaries, letters; protocols; pre-prints; case reports and series n < 20; guidelines
Timeframe	1 January 2021 to 26 June 2026	Published before 1 January 2021
Language and country	No language restriction and no geographical or country restriction were applied at any stage; studies from any country were eligible	None

The five-year date restriction was applied because array resolution and, more importantly, CNV classification practice changed materially over the preceding decade. The ACMG/ClinGen evidence-weighted scoring framework that most contemporary laboratories now apply was published only in 2020 [5], and yields reported under earlier, laboratory-specific classification schemes are not directly comparable with those reported under it, since the threshold for calling a variant pathogenic differs. Pooling pre- and post-2020 cohorts would therefore have introduced a source of heterogeneity that no statistical adjustment could remove. The trade-off--a smaller evidence base--is acknowledged among the limitations.

Information Sources and Search Strategy

PubMed/MEDLINE, Embase, Scopus, and the Web of Science Core Collection were searched from 1 January 2021 to 26 June 2026, which was the date of the last search. Concept blocks combined array-based testing terms, neurodevelopmental phenotype terms, yield and copy number terms, and pediatric terms, using database-appropriate syntax; the full search strings and per-database record counts are provided in the Appendix (see Table 7). Because requiring yield-related terminology in the title or abstract could, in principle, miss eligible cohorts that report yield only in the full text, search sensitivity was checked informally against a seed set of studies known in advance to be eligible; all seed studies were retrieved by the executed strings. This check was not a formal relative-recall analysis, and residual insensitivity cannot be excluded. Reference lists of all included articles and of recent relevant reviews were hand-searched, and forward citation tracking was performed in Google Scholar; these supplementary methods identified no additional eligible records.

Study Selection

Retrieved records were exported to a reference manager and de-duplicated, after which two reviewers independently screened titles and abstracts and then assessed full texts against the eligibility criteria, with disagreements resolved by discussion and recourse to a third reviewer where required. The search identified 434 records (PubMed: 202, Embase: 88, Scopus: 73, Web of Science: 71), of which 267 duplicates were removed, leaving 167 records for screening. Eighty-three records were excluded at title and abstract screening; of the 84 reports sought for retrieval, one could not be obtained because of a paywall, leaving 83 reports assessed in full for eligibility. Seventy reports were excluded--34 based on adult populations, 23 abstracts, editorials, or review articles, and 13 not reporting patient-level diagnostic yield--leaving 13 studies for inclusion in the qualitative synthesis (Figure 1). No record was excluded on the grounds of being a preprint.

**Figure 1:**
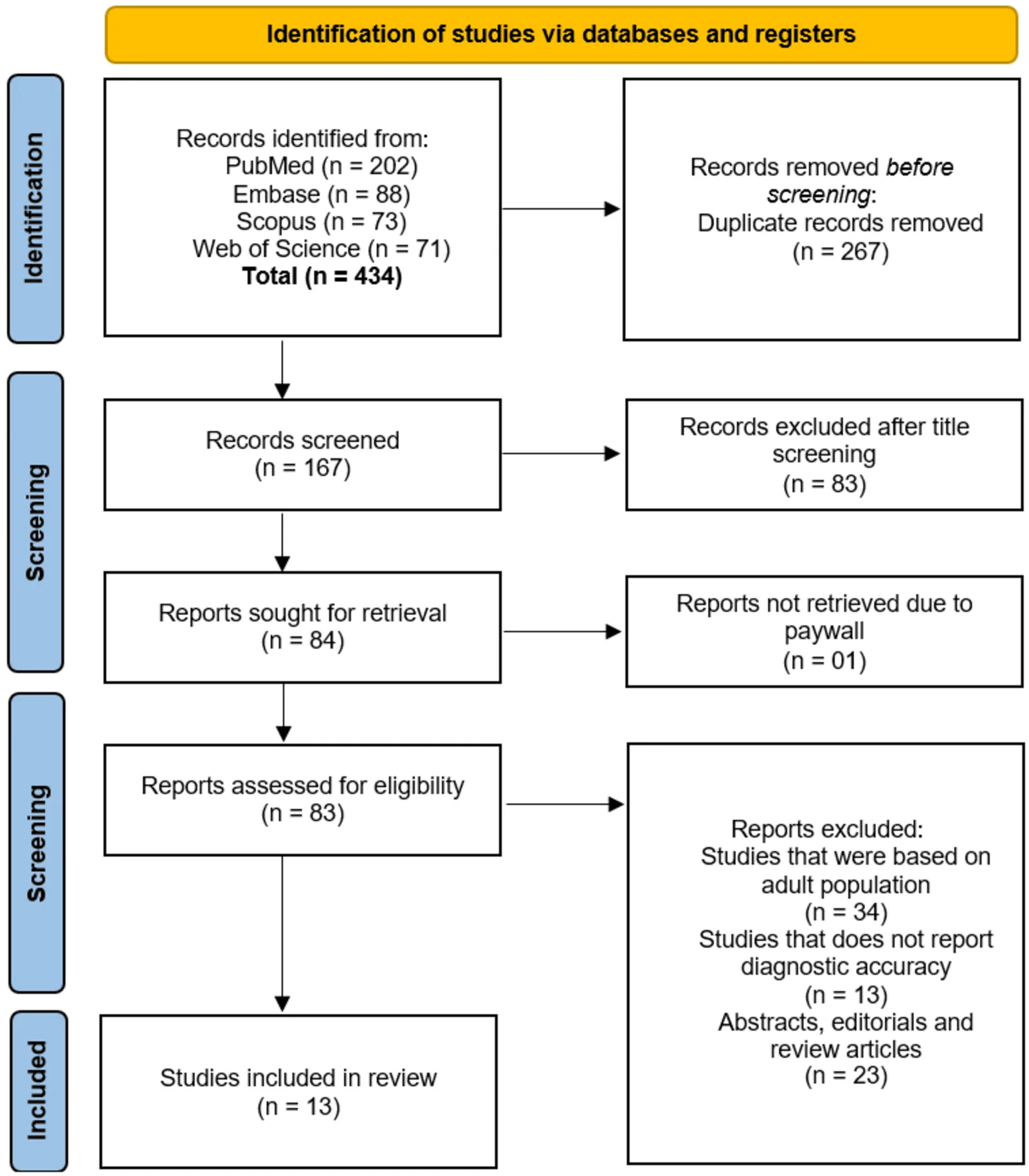
PRISMA 2020 flow diagram of study identification, screening, and inclusion. PRISMA: Preferred Reporting Items for Systematic Reviews and Meta-Analyses.

Data Extraction

A standardized, pre-piloted form was used to extract first author, year, country and center, study design and recruitment period, ascertainment setting, sample size, age and sex distribution, referral indication and phenotypic composition, array platform and nominal resolution, CNV classification framework, the number and proportion of participants with pathogenic or likely pathogenic CNVs, the proportion with variants of uncertain significance (VUS), phenotype-stratified yields, inheritance data, comparative karyotype yield, and any reported downstream clinical consequence. Data were extracted independently in duplicate and cross-checked against the source article. The primary outcome was patient-level diagnostic yield as defined above; secondary outcomes were the VUS rate, phenotype-stratified yield, and clinical utility, defined a priori as any documented change in surveillance, medication, specialist referral, or reproductive or family-planning counseling attributable to the CMA result.

Assessment of Formal Diagnostic Performance Metrics and Predictive Modeling

Because the value of any diagnostic test is conventionally expressed through accuracy metrics rather than yield alone, the extraction form additionally captured, for every included study, whether the authors reported sensitivity, specificity, positive or negative predictive value, area under the receiver operating characteristic curve, calibration slope or intercept, derivation and validation cohort sizes, internal or external validation, or comparative performance against karyotype or another standard clinical pathway. The same form captured whether any study developed a machine-learning or multivariable prediction model and, if so, whether it addressed overfitting, data leakage, class imbalance, feature-to-sample ratio, nested cross-validation, external validation, model interpretability, and calibration. Had any such model been identified, it would have been appraised using a prediction model risk-of-bias instrument (PROBAST) in addition to the case-series checklist.

Risk-of-Bias Assessment

All 13 included studies shared the same underlying architecture: a single arm of consecutively or non-consecutively referred patients, all of whom received the index test, with no control group, no independent reference standard, and no comparative allocation. Although the authors variously described their cohorts as retrospective cohorts, laboratory cohorts or case series, this is a difference of label rather than of design, and instruments built for comparative diagnostic accuracy studies, such as QUADAS-2, cannot be applied because there is no reference standard against which CMA could be indexed. The Joanna Briggs Institute (JBI) critical appraisal checklist for case series was therefore used throughout, as it is validated for precisely this single-arm architecture and its ten items map directly onto the threats to validity that operate in referral-based yield studies [12]. Applying one instrument to all studies also keeps the appraisal internally comparable.

Each item was rated Yes, No, or Unclear by two reviewers independently, with disagreements resolved by consensus. To avoid the reproducibility problem inherent in holistic judgment, item-level ratings were converted into an overall category using rules fixed before appraisal began. A study was rated at low risk of bias when at least eight of the ten items were rated Yes and both Q4 (consecutive inclusion) and Q5 (complete inclusion) were rated Yes. It was rated at high risk when either Q4 or Q5 was rated No, or when three or more items in total were rated No or Unclear. All remaining studies were rated at moderate risk. Q4 and Q5 were given this decisive weight because non-consecutive or incomplete ascertainment is the mechanism by which diagnostic yield is most readily inflated in referral-based cohorts, and no other item can compensate for it.

Data Synthesis

Findings were synthesized narratively and presented in tabular form. A random-effects meta-analysis of proportions was considered and would have been technically feasible, and we do not suggest that heterogeneity in itself makes pooling impossible. It was not undertaken because the numerator was not defined identically across cohorts--some counted uniparental disomy or aneuploidy within the diagnostic yield and others did not--so a pooled estimate would have aggregated quantities that are not the same quantity, a problem that random-effects modeling accommodates but does not solve. Study-level yields are therefore reported individually with exact numerators and denominators, together with Wilson score 95% confidence intervals calculated by the review team from the reported counts. The summary value of 18.5% is the unweighted median of the 13 study-level yields and is described as such throughout; it is not a pooled or population-weighted estimate, and because cohorts ranged from 78 to 5,778 participants, it gives the smallest and largest studies equal influence. Subgroup patterns were examined descriptively. Effect estimates reported by a single cohort are identified as such and are not presented as generalizable across the evidence base.

Results

Study Characteristics

Thirteen studies published between 2021 and 2026 fulfilled the eligibility criteria [13-25], together reporting CMA results for 11,535 children from nine countries, with individual cohorts ranging from 78 [17] to 5,778 participants [15]. The studies originated from South Korea [13], China [14], Brazil [15,22], Serbia [16], Hungary [17], Romania [18,19], India [20,24], Italy [21], and Türkiye [23,25]. All were retrospective analyses of clinically referred cohorts drawn from tertiary pediatric, medical genetics, or diagnostic laboratory settings, and in all of them, CMA was performed as part of routine diagnostic care rather than within a research protocol. Full characteristics are presented in Table 2.

**Table 2:** Characteristics of the included studies. ADHD, attention-deficit/hyperactivity disorder; ASD, autism spectrum disorder; CNV, copy number variant; DD, developmental delay; GDD, global developmental delay; ID, intellectual disability; LCSH, long contiguous stretches of homozygosity; MCA, multiple congenital anomalies; NDD, neurodevelopmental disorder; pCNV, pathogenic copy number variant; pp, percentage points; UPD, uniparental disomy; VOUS/VUS, variant of uncertain significance; ROH, regions of homozygosity.

Author, year (Ref)	Country	Design	Population/indication	n	Platform	Comparator	Outcomes reported	Key findings
Yang et al., 2021 [13]	South Korea	Retrospective cohort (2010-2020)	Unexplained DD/ID; ASD 12.0%, epilepsy 11.0%, ADHD 3.9%	308	Affymetrix CytoScan 750K (~100 kb)	None	Yield; CNV size, gene content, type; phenotype associations	Yield 18.5% (57/308). Pathogenicity tracked gene content and deletion type, not CNV size.
Liu et al., 2022 [14]	China	Retrospective cohort	ASD (n=151) and unexplained ID/DD (n=259)	410	Affymetrix CMA after karyotype	G-banded karyotype	Yield by phenotype, sex and age; de novo rate	Yield 22.4% (20.9% excl. aneuploidy). ASD 3.3%, ID/DD 31.5%. Female 31.3% vs male 16.1% (p=4.8×10⁻⁴).
Krepischi et al., 2022 [15]	Brazil	Retrospective laboratory cohort (2010–2020)	NDD and/or congenital anomalies without evident cause	5,778	Illumina CytoSNP 850K; Affymetrix 750K (n=810)	None	Yield; CNV class distribution; UPD/ROH; VUS segregation	Yield 19.7%. 22q11.2 deletion commonest (14.6%). Parental testing of 126 VUS reclassified only 1.
Perovic et al., 2022 [16]	Serbia	Retrospective cohort	Congenital anomalies, DD, ID, ASD, epilepsy	430	Oligonucleotide array-CGH	None	Detection rate overall and by phenotypic category	Yield 16.3% (70/430); any non-benign CNV 26.5%; VUS 6.7%. Yield rose with phenotypic complexity.
Lengyel et al., 2022 [17]	Hungary	Retrospective case series	NDD and/or congenital anomalies referred for genetic testing	78	Multiple CMA platforms	None	Yield; phenotype-genotype comparison; VUS characterization	Yield 37.2% (29/78); 24 VUS in 17 patients. No phenotypic feature reached significance.
Miclea et al., 2022 [18]	Romania	Retrospective cohort	GDD/ID after full clinical, metabolic and karyotype evaluation	189	CMA (post-karyotype)	Karyotype	Pathogenic CNV and UPD rate; recurrent CNV share	Pathogenic CNV/UPD 18.51% (35/189); with VUS 28.04%. Two UPD15 cases. Recurrent CNVs 60% of positives.
Streață et al., 2022 [19]	Romania	Retrospective multi-center cohort (2015-2022)	Syndromic and non-syndromic GDD/ID without epilepsy; median age 5.5 y	371	Agilent and Oxford Gene Technology	Karyotype (subset)	Yield by ID severity and phenotype	Yield 21.29% (79/371). Facial dysmorphism 22.97%; ASD 19.11%; GDD/ID + MCA 27.42%.
Kamath et al., 2022 [20]	India	Retrospective cohort	Unexplained GDD/ID with and without co-occurring conditions	81	CMA	Prior karyotype (n=6)	Pathogenic CNV yield; phenotypic predictors	Yield 20.9%; 17 pCNVs (13 losses, 4 gains). Cardiac defect the only significant predictor in this cohort (OR 6.13, p=0.031).
Cucinotta et al., 2023 [21]	Italy	Retrospective cohort, standardized phenotyping	Children and young people with ASD	329	Array-CGH	None	Yield; enriched gene sets	Yield 15.2% (50/329), within the previously reported ASD range of 9.3-29%.
Chaves et al., 2024 [22]	Brazil	Retrospective laboratory cohort	NDD and/or congenital anomalies; ASD in ~one third (sub-cohort)	1,012	Affymetrix CytoScan HD (41%) and 750K (59%)	None	Yield overall and in ASD; VUS rate; LCSH analysis	Yield 17%. ASD 10% (16% with dysmorphism, 7% isolated). VUS-only 12%. LCSH ≥3 Mb in 91%.
Akkus and Cubuk, 2024 [23]	Türkiye	Retrospective cohort	Unexplained DD/ID, ASD, and/or multiple congenital anomalies	1,227	CMA	None	Phenotype-associated etiological diagnosis rate	Phenotype-associated diagnosis in 135 patients (11.0%).
Lall et al., 2026 [24]	India	Retrospective cohort	DD, dysmorphism, autism, ID, or congenital malformations	300	Agilent 4×180K	Karyotype in all 300	CMA vs karyotype yield; VOUS rate	CMA 16.0% vs karyotype 7.0% (+9.0 pp). VOUS 2.66%.
Sanri et al., 2026 [25]	Türkiye	Retrospective cohort	DD, ID, ASD, epilepsy, and/or congenital anomalies	1,022	CMA, ACMG classification	None	Yield; CNV class and type distribution	Any CNV 27.3% (279/1,022); pathogenic/likely pathogenic 14.8% (151/1,022). Deletions more often pathogenic.

No cohort from the United States, the United Kingdom, Germany, or any other high-income Western setting met the eligibility criteria, and because this pattern could be mistaken for a design decision, it warrants explicit comment: no geographical or national restriction was applied at any stage of the search or screening, and no study was excluded on the basis of its country of origin. The distribution appears instead to reflect where CMA remains the principal first-line genomic test. In health systems where exome or genome sequencing is now recommended as a first- or second-tier investigation and is reimbursed accordingly [8], contemporary cohort publications increasingly report sequencing yields, with CMA appearing as a comparator or a legacy test rather than as the subject of the study; single-center CMA yield series from those settings were largely published before the 2021 date limit. The consequence for interpretation is important and is revisited among the limitations: these findings describe CMA performance in settings that still depend on it and should not be assumed to transfer unchanged to Western European or North American referral populations, which differ in ancestry, consanguinity rates, and pre-test triage pathways.

Array platforms varied. Affymetrix CytoScan 750K or CytoScan HD arrays were used in the Korean [13], Chinese [14], and southern Brazilian [22] cohorts; the São Paulo cohort used the Illumina Infinium CytoSNP 850K BeadChip with a subset on Affymetrix CytoScan 750K [15]; and Agilent or Oxford Gene Technology oligonucleotide platforms were used in the Romanian [19] and Indian [24] cohorts. Studies published from 2022 onward generally classified variants against ACMG or ACMG/ClinGen criteria, whereas earlier or laboratory-specific frameworks were applied elsewhere, contributing to heterogeneity in the pathogenic threshold.

Overall Diagnostic Yield

Study-level diagnostic yield of pathogenic and likely pathogenic CNVs ranged from 11.0% (95% CI 9.4-12.9) to 37.2% (95% CI 27.3-48.3), with an unweighted median across the 13 cohorts of 18.5% (Table 3). Eleven of the 13 studies reported a yield between 14% and 23% [13-16,18-22,24,25], clustering around the figure anticipated by the original first-tier consensus [4]; only the Hungarian [17] and one Turkish [23] cohort fell outside this band. The confidence intervals make the apparent precision of this clustering less impressive than the point estimates alone suggest: the intervals for the smaller cohorts are wide, spanning 13.5-31.1% in the Indian GDD/ID series [20] and 27.3-48.3% in the Hungarian series [17], and most of the intervals for the mid-sized cohorts overlap one another substantially.

**Table 3:** Diagnostic performance of CMA across included studies. NA, not applicable; NR, not reported; P/LP, pathogenic/likely pathogenic; pp, percentage points; UPD, uniparental disomy; VUS, variant of uncertain significance; NS, not significant; ID, intellectual disability; DD, developmental delay; ASD, autism spectrum disorder; GDD, global developmental delay; CNV, copy number variant; MCA, multiple congenital anomalies; CMA, chromosomal microarray analysis.

Author, year (Ref)	n	Overall yield (P/LP ± UPD)	95% CI	ASD phenotype	DD/ID phenotype	Syndromic/complex phenotype	VUS rate	Karyotype comparison
Yang et al., 2021 [13]	308	18.5% (57/308)	14.6-23.2	NR	18.5% (Whole cohort DD/ID)	NR	NR	NR
Liu et al., 2022 [14]	410	20.9% (84/402) excl. aneuploidy; 22.4% incl.	17.2-25.1	3.3% (5/151)	31.5% (79/251); 33.6% (87/259) incl. aneuploidy	NR	NR	Karyotype performed first
Krepischi et al., 2022 [15]	5,778	19.7% (P+LP+UPD)	18.7-20.7†	NR	NR	NR	40% of 2,270 relevant CNVs (771 individuals)	NR
Perovic et al., 2022 [16]	430	16.3% (70/430)	13.1-20.1	NR	Highest where DD/ID prevailing	"Dysmorphism plus" highest; isolated anomalies lowest	6.7% (29/430)	NR
Lengyel et al., 2022 [17]	78	37.2% (29/78)	27.3-48.3	NR	NR	GDD trend p=0.081 (NS)	24 VUS in 17 patients	NR
Miclea et al., 2022 [18]	189	18.51% (35/189, incl. UPD)	13.6-24.7	NR	18.51% (whole cohort GDD/ID)	NR	Pathogenic + VUS 28.04%	Karyotype in all
Streață et al., 2022 [19]	371	21.29% (79/371)	17.4-25.7	19.11% (26/136)	Mild ID 17.05%; moderate/severe 23.55%	Dysmorphism 22.97%; finger anomalies 20%; GDD/ID+MCA 27.42%	NR	Karyotype as alternative
Kamath et al., 2022 [20]	81	20.9% (17/81)	13.5-31.1	NR	20.9% (whole cohort GDD/ID)	Cardiac defect OR 6.13 (p=0.031), this cohort only	NR	Abnormality in six previously karyotyped
Cucinotta et al., 2023 [21]	329	15.2% (50/329)	11.7-19.5	15.2% (whole cohort ASD)	NA	NR	NR	NR
Chaves et al., 2024 [22]	1,012	17% (206 pathogenic CNVs)	14.8-19.4†	10%; 16% with dysmorphism; 7% isolated	NR	16% for ASD with dysmorphic features	12% VUS-only	NR
Akkus & Cubuk, 2024 [23]	1,227	11.0% (135/1,227)	9.4-12.9	NR	NR	NR	NR	NR
Lall et al., 2026 [24]	300	16.0% (48/300)	12.3-20.6	NR	NR	NR	2.66% (8/300)	7.0% (21/300), 95% CI 4.6-10.5
Sanri et al., 2026 [25]	1,022	14.8% (151/1,022)	12.7-17.1	NR	NR	NR	Any CNV 27.3%	NR
Summary	11,535	Range 11.0-37.2%; median study-level yield 18.5%	-	Range 3.3-15.2%	Range 17.05-33.6%	Up to 27.42%	Range 2.66-12%	CMA increment +9.0 pp in the single head-to-head cohort

The Korean cohort of 308 children identified 70 pathogenic CNVs among 221 detected variants, giving a yield of 18.5% in 57 patients (95% CI 14.6-23.2) [13]. In the Chinese cohort, the yield was 22.4% when the eight aneuploidies were included and 20.9% (84/402, 95% CI 17.2-25.1) when they were excluded [14]. The São Paulo cohort of 5,778 patients, the largest included, reported an overall yield of 19.7% once pathogenic CNVs, likely pathogenic CNVs and uniparental disomies were counted [15], while the Serbian cohort detected 140 non-benign CNVs in 113 of 430 individuals (26.5%), of which clinically significant variants in 70 patients produced a yield of 16.3% (95% CI 13.1-20.1) [16].

Two independently reported Romanian cohorts gave concordant results: 35 of 189 patients (18.51%, 95% CI 13.6-24.7) carried pathogenic CNVs or uniparental disomy in the Cluj-Napoca series [18], and 79 of 371 patients (21.29%, 95% CI 17.4-25.7) carried pathogenic or likely pathogenic CNVs in the Craiova series [19]. The Indian GDD/ID cohort reported a pathogenic CNV yield of 20.9%, with 17 pathogenic CNVs comprising 13 losses and four gains [20], and the southern Brazilian cohort identified 206 pathogenic CNVs in 17% of 1,012 participants (95% CI 14.8-19.4) [22]. The two Turkish cohorts sat at the lower end of the range: a phenotype-associated etiological diagnosis was established in 135 of 1,227 patients (11.0%) in one [23], and in the other, although CNVs were identified in 279 of 1,022 patients (27.3%), only 151 (14.8%, 95% CI 12.7-17.1) carried a pathogenic or likely pathogenic variant [25]. The New Delhi cohort reported 16.0% (48/300, 95% CI 12.3-20.6) [24] and the Italian ASD cohort 15.2% (50/329, 95% CI 11.7-19.5) [21]. The single high outlier was the Hungarian series, in which 30 pathogenic CNVs were identified in 29 of 78 children (37.2%) [17]; this was the smallest cohort included and was assembled at a time when CMA was not part of routine genetic testing in Hungary, so that children were selected for testing on the basis of a high pre-test probability of a chromosomal aetiology--an ascertainment pattern that plausibly accounts for the elevated yield.

Formal Accuracy Metrics, Comparative Performance, and Predictive Modeling

None of the 13 included studies reported sensitivity, specificity, positive or negative predictive value, area under the receiver operating characteristic curve, or any measure of calibration, and none developed or validated a prediction model. Table 4 records this systematically and, for each metric, states why it was not estimable from this evidence base rather than simply noting its absence. The central reason is structural: every included study applied CMA as the sole index test without an independent reference standard, so there is no external classification of true disease status against which array results could be cross-tabulated. Diagnostic yield is therefore a measure of detection under routine referral conditions, not of accuracy, and the two should not be used interchangeably.

Comparative performance against standard clinical testing was reported by only one cohort. Lall et al. performed both karyotyping and CMA in all 300 participants and found a CMA yield of 16.0% (48/300, 95% CI 12.3-20.6) against a karyotype yield of 7.0% (21/300, 95% CI 4.6-10.5) [24]. Because the paired discordant counts required for a McNemar test or a paired confidence interval were not reported, the 9.0 percentage-point difference cannot be given a valid paired interval; an unpaired Newcombe interval of approximately 3.9 to 14.2 percentage points is provided only as an indication of magnitude and is conservative for paired data. This is a single comparison in a single center, and it establishes that CMA detected more abnormalities than karyotype in those patients, not that CMA is more accurate in any formal sense.

No included study applied machine learning, multivariable risk prediction, or any high-dimensional classifier to CMA or phenotypic data. In consequence, the methodological concerns that properly attach to such analyses--overfitting, data leakage between derivation and validation partitions, class imbalance, an unfavorable feature-to-sample ratio, the need for nested cross-validation, external validation, model interpretability and calibration--did not arise as appraisal domains for any study in this review, and no prediction-model risk-of-bias instrument was indicated. This is itself a finding worth recording because the phenotypic determinants summarized in Table 4 are exactly the kind of variables that a properly validated prediction model could combine into a calibrated pre-test probability, and no such model yet exists for this population.

**Table 4:** Formal diagnostic performance metrics: reporting across the 13 included studies and reasons for non-estimability. ROC, receiver operating characteristics; PROBAST, Prediction model Risk Of Bias ASsessment Tool; CMA, chromosomal microarray analysis; CNV, copy number variant; VUS, variant of uncertain significance.

Performance domain	Reported by any included study?	Why it could not be assessed in this evidence base
Sensitivity/specificity	No (0/13)	Estimation requires an independent reference standard classifying each child as truly affected or unaffected by a CNV-detectable disorder. No included study applied one; CMA was both the index test and, in effect, the only test.
Positive/negative predictive value	No (0/13)	Requires the above plus a defined pre-test population; the referral-based, non-consecutive ascertainment of these cohorts makes prevalence undefined.
Area under the ROC curve (AUC)	No (0/13)	Requires a continuous or ranked predictor score. CMA reporting is categorical (pathogenic/likely pathogenic/VUS/likely benign/benign) and no study derived a risk score.
Calibration (calibration slope, intercept, plots)	No (0/13)	Applies to predicted probabilities from a model. No included study produced predicted probabilities.
Validation-cohort size; internal or external validation	No (0/13)	No included study developed a prediction model, so no derivation or validation cohorts existed; none reported nested cross-validation or an external validation set.
Comparative performance versus standard clinical testing	Partially (1/13)	Only Lall et al. [24] tested both CMA and karyotype in all participants: CMA 16.0% (95% CI 12.3-20.6) versus karyotype 7.0% (95% CI 4.6-10.5). The paired discordant counts needed for a McNemar test were not reported, so the difference is presented descriptively.
Machine-learning or multivariable prediction modeling	No (0/13)	No included study applied machine learning, multivariable risk prediction, or any high-dimensional classifier. Consequently, overfitting, data leakage, class imbalance, feature-to-sample ratio, nested cross-validation, interpretability, and model calibration were not applicable appraisal domains, and no prediction-model risk-of-bias tool (e.g., PROBAST) was indicated.

Phenotype and Other Determinants of Yield

Every study that stratified yield by phenotype found the same directional relationship: the more complex the phenotype, the higher the diagnostic yield, and isolated ASD was consistently the lowest-yield indication. Because this ordering is easily illustrated by a single striking example but is more persuasive when documented across the whole evidence base, every determinant examined in every included cohort is set out in Table 5, including the cohorts in which no determinant reached significance and the one cohort that reported no determinant analysis at all.

**Table 5:** Determinants of diagnostic yield examined and reported in each included study. ASD, autism spectrum disorder; CNV, copy number variant; GDD, global developmental delay; ID, intellectual disability; LCSH, long contiguous stretches of homozygosity; MCA, multiple congenital anomalies; OR, odds ratio; UPD, uniparental disomy.

Author, year (Ref)	Determinant(s) examined	Reported finding
Yang et al., 2021 [13]	CNV gene content; CNV type; CNV size; associated clinical features	Pathogenicity correlated with the number of genes within the CNV and with a deletion copy-number state, but not with CNV size. Short stature and hearing difficulty were more frequent in the pathogenic CNV group.
Liu et al., 2022 [14]	Phenotype (ASD vs ID/DD); sex; inheritance	ASD 3.3% (5/151) vs ID/DD 31.5% (79/251). Female 31.3% (40/128) vs male 16.1% (44/274), p = 4.8 × 10⁻⁴. De novo CNV in 14.9% (60/402).
Krepischi et al., 2022 [15]	Recurrent syndromic loci; second-hit burden	477 patients (46.6% of pathogenic cases) carried one of 71 recognized microdeletion/microduplication syndromes; commonest 22q11.2 deletion (14.6%), 15q13.3 duplication (6.6%), 16p11.2 deletion (5%), and 15q11.2 deletion (4.8%). A second CNV hit was present in 26.4% of susceptibility-CNV carriers.
Perovic et al., 2022 [16]	Phenotypic complexity category	Detection rate of clinically significant CNVs rose with phenotypic complexity; the "dysmorphism plus" category was highest and isolated congenital anomalies lowest.
Lengyel et al., 2022 [17]	Individual phenotypic features (n = 78)	No feature reached statistical significance. Non-significant trends: postnatal growth delay (p = 0.056), pectus excavatum (p = 0.075), brain imaging abnormality (p = 0.078), global developmental delay (p = 0.081), macrocephaly (p = 0.089). The series also reported phenotypic expansion of the 14q11.2 microdeletion (SUPT16H, CHD8).
Miclea et al., 2022 [18]	Recurrent vs non-recurrent CNV; UPD	Recurrent CNVs accounted for 21 of 35 pathogenic findings (60%). Two patients had UPD15 (Prader-Willi and Angelman phenotypes).
Streață et al., 2022 [19]	ID severity; facial dysmorphism; ASD; finger anomalies; multiple congenital anomalies	Mild ID 17.05% (22/129) vs moderate-to-severe ID 23.55% (57/242). Facial dysmorphism 22.97% (71/309); ASD 19.11% (26/136); finger anomalies 20% (27/96); GDD/ID with MCA 27.42% (17/62).
Kamath et al., 2022 [20]	Co-occurring cardiac defect and other comorbidities	Co-occurring cardiac defect was the only phenotypic variable significantly associated with a pathogenic CNV in this cohort (OR 6.13, p = 0.031); other comorbidities were not significant.
Cucinotta et al., 2023 [21]	ASD with standardized phenotypic and psychopathological characterization	Yield 15.2% (50/329) in a comprehensively phenotyped ASD cohort, at the upper end of previously reported ASD estimates.
Chaves et al., 2024 [22]	ASD with/without dysmorphism or ID; long contiguous stretches of homozygosity	Isolated ASD 7%; ASD overall 10%; ASD with dysmorphic features 16%. LCSH ≥3 Mb in 91% of 953 arrays; pattern suggesting first- to fifth-degree consanguinity in ~11.5%; putative UPD in 2.8%.
Akkus and Cubuk, 2024 [23]	Not reported separately	No phenotype-stratified or variant-level determinant analysis was reported.
Lall et al., 2026 [24]	Test modality (CMA vs karyotype, same patients)	CMA 16.0% (48/300) vs karyotype 7.0% (21/300); absolute increment 9.0 percentage points.
Sanri et al., 2026 [25]	CNV type	CNVs were more frequently classified as pathogenic when they presented as deletions rather than duplications.

The clearest single demonstration came from the Chinese cohort, where yield was 3.3% (5/151) in ASD but 31.5% (79/251) in ID/DD after exclusion of aneuploidies, rising to 33.6% (87/259) when they were included [14]. The southern Brazilian ASD sub-cohort showed the same gradient within a single phenotype: the overall ASD diagnostic rate was 10%, rising to 16% when ASD was accompanied by dysmorphic features and falling to 7% for isolated ASD without ID or dysmorphism [22]. The Italian ASD-only cohort reported 15.2%, at the upper end of the ASD range and consistent with a cohort that underwent comprehensive phenotypic and psychopathological characterization before testing [21]. In the Serbian cohort, the more complex the phenotype, particularly where DD/ID was the prevailing feature, the higher the detection rate of clinically significant CNVs, with isolated congenital anomalies lowest and the "dysmorphism plus" category highest [16]. The Craiova cohort quantified this in detail: yield was 22.97% (71/309) where facial dysmorphism accompanied GDD/ID, 19.11% (26/136) where ASD was present, 20% (27/96) where finger anomalies were present, and 27.42% (17/62) where GDD/ID occurred with multiple congenital anomalies, while yield was comparable between mild ID (17.05%, 22/129) and moderate-to-severe ID (23.55%, 57/242) [19]. In the Indian GDD/ID cohort, a co-occurring cardiac defect was the only phenotypic variable significantly associated with a pathogenic CNV (odds ratio 6.13, p = 0.031) [20]; this estimate derives from a single cohort of 81 children, was not examined in any other included study, was not adjusted for multiple comparisons, and should be read as a hypothesis-generating finding rather than an established predictor.

Determinants beyond phenotype were reported by two cohorts [13,25]. The Korean cohort found that CNV pathogenicity correlated with the number of genes contained within the CNV interval and with a deletion rather than duplication copy-number state, but not with CNV size, and that short stature and hearing difficulty were more frequent in the pathogenic CNV group [13]; the Turkish cohort of 1,022 children likewise found CNVs more frequently classified as pathogenic when they presented as deletions [25]. The Chinese cohort reported a higher yield in females (31.3%, 40/128) than in males (16.1%, 44/274) across all neurodevelopmental disorders (p = 4.8 × 10⁻⁴), interpreted by the authors as evidence for a female protective mechanism, with a de novo CNV identified in 14.9% (60/402) of that cohort [14]; no other included cohort reported sex-stratified yield, so this observation is unreplicated within the present evidence base. In the Hungarian series, no phenotypic predictor reached statistical significance in a cohort of 78, although postnatal growth delay, pectus excavatum, brain imaging abnormalities, global developmental delay, and macrocephaly all showed non-significant trends [17].

Consistency and Inconsistency Across Studies

The direction of the phenotype effect was consistent in every cohort that examined it, and no included study reported a yield in isolated ASD that approached the yield in DD/ID with congenital anomalies; on this point, the evidence base contains no contradiction. Three genuine inconsistencies were nonetheless identified. First, the two Turkish cohorts, drawn from broadly comparable referral populations, reported yields of 11.0% and 14.8% [23,25], and the lower of these was defined as a phenotype-associated etiological diagnosis rather than as any pathogenic CNV, so part of this gap is definitional rather than biological. Second, the assumption that yield rises with the severity of intellectual disability was not supported in the only cohort to test it directly, which found 17.05% in mild ID against 23.55% in moderate-to-severe ID with overlapping ranges [19]. Third, the female excess in yield reported in the Chinese cohort [14] was neither replicated nor contradicted, because no other cohort was stratified by sex. Beyond these, the principal inconsistency across the evidence base is not in the findings but in the definitions: whether uniparental disomy and aneuploidy were counted within the numerator varied between cohorts and shifts reported yields by one to two percentage points independently of any real difference in detection.

Variants of Uncertain Significance and Interpretive Burden

The VUS burden was reported inconsistently but was substantial wherever it was quantified. The Serbian cohort reported VUS in 29 of 430 patients (6.7%) alongside likely benign variants in 14 (3.2%) [16]; the New Delhi cohort reported VOUS in 2.66% (8/300) and described them as challenging to interpret [24]; the southern Brazilian cohort found that 12% of patients carried a rare variant of uncertain clinical significance as their only clinically relevant finding [22]; and the Hungarian series identified 24 VUS in 17 of 78 patients [17]. The Romanian Cluj cohort reported that pathogenic findings and VUS combined were present in 53 of 189 patients (28.04%) against a pathogenic-only yield of 18.51%, so that roughly a third of all abnormal results were uninterpretable at the time of reporting [18]. The São Paulo cohort addressed the downstream cost of VUS directly: among 126 patients with an autosomal VUS in whom parental segregation was tested, 125 variants proved to be inherited and only one was reclassified, so that 252 parental CMA tests yielded a single actionable reclassification, leading the authors to conclude that in resource-constrained settings routine parental CMA to resolve VUS segregation offers poor cost-benefit relative to testing additional probands [15].

Comparison With Karyotype and Additional Array-Derived Findings

Two studies provided direct within-cohort comparison against conventional cytogenetics [20,24], and the quantitative comparison is reported above. In the Indian GDD/ID cohort, six children karyotyped before CMA showed a structural abnormality, and CMA provided additional characterizing information in these previously karyotyped patients [20]. SNP-based platforms also yielded findings beyond copy number. The southern Brazilian cohort analyzed long contiguous stretches of homozygosity (≥3 Mb) in 953 arrays and detected at least one such region in 91% of patients, with the pattern suggesting consanguinity of first- to fifth-degree in approximately 11.5% and a putative uniparental disomy in 2.8% [22]. The São Paulo cohort detected copy-neutral regions of homozygosity in 259 patients, of which 26 were classified as uniparental disomy and 14 were pathogenic by virtue of mapping to imprinted chromosomes, with UPD15 the most frequent [15]; the Cluj cohort similarly identified two patients with UPD15 presenting with Prader-Willi and Angelman syndrome phenotypes [18]. Recurrent CNVs accounted for a large share of positive results, being seen in 21 of 35 patients with pathogenic findings (60%) in the Cluj cohort [18], while in the São Paulo cohort 477 patients (46.6% of pathogenic cases) carried CNVs associated with 71 distinct microdeletion or microduplication syndromes, most frequently 22q11.2 deletion (14.6%), 15q13.3 duplication encompassing CHRNA7 (6.6%), 16p11.2 deletion (5%), 15q11.2 deletion (4.8%) and Prader-Willi/Angelman region variants (4.6%), and a second CNV hit was identified in 26.4% of patients carrying a susceptibility CNV of incomplete penetrance [15].

Clinical Utility

The second review question can be answered briefly, because the contemporary evidence is almost silent. None of the 13 included studies prospectively documented a change in surveillance, medication, specialist referral, or reproductive counseling attributable to the CMA result, and none reported a clinically actionable yield distinct from the diagnostic yield. Diagnostic yield was the terminal reported outcome in every cohort. This is a finding about the literature rather than about the test, and it is treated as such in the Discussion.

Risk of Bias

Risk-of-bias assessment is presented in Table 6, applying the pre-specified decision rules described in the Methods. Seven studies were judged at low risk of bias [13,14,16,19,21,24,25], two at moderate risk [18,22], and four at high risk [15,17,20,23]. Inclusion criteria were clearly stated, and the condition was identified by valid methods in all 13 studies. The seven low-risk cohorts each satisfied both Q4 and Q5 and at least eight items overall. The four high-risk judgements arose in three distinct ways under the rules: the São Paulo and Turkish laboratory-derived series each accumulated three or more Unclear or No ratings, principally for demographic and clinical reporting and for enrollment [15,23]; the Hungarian series was rated No on both Q4 and Q5, reflecting selective referral in a setting where CMA was not yet routine [17]; and the Indian GDD/ID cohort was Unclear on enrollment, demographic reporting and outcome completeness, again reaching the three-item threshold [20]. Across the evidence base as a whole, no study reported an independent blinded reference standard, and referral-based ascertainment remains the principal structural threat to the generalizability of the yields reported.

**Table 6:** Risk of bias of included studies (JBI critical appraisal checklist for case series) Q1, clear inclusion criteria; Q2, standard and reliable condition measurement; Q3, valid identification methods; Q4, consecutive inclusion; Q5, complete inclusion; Q6, clear demographic reporting; Q7, clear clinical reporting; Q8, clear outcome reporting; Q9, clear site/clinic reporting; Q10, appropriate statistical analysis. Overall category assigned by pre-specified rules: Low = ≥8 items Yes and both Q4 and Q5 Yes; High = Q4 or Q5 rated No, or ≥3 items rated No/Unclear; Moderate = all others. JBI, Joanna Briggs Institute; RoB, risk of bias.

Author, year (Ref)	Q1	Q2	Q3	Q4	Q5	Q6	Q7	Q8	Q9	Q10	Overall RoB
Yang et al., 2021 [13]	Yes	Yes	Yes	Yes	Yes	Yes	Yes	Yes	Yes	Yes	Low
Liu et al., 2022 [14]	Yes	Yes	Yes	Yes	Yes	Yes	Yes	Yes	Yes	Yes	Low
Krepischi et al., 2022 [15]	Yes	Unclear	Yes	Unclear	Unclear	No	No	Yes	Yes	Yes	High
Perovic et al., 2022 [16]	Yes	Yes	Yes	Yes	Yes	Yes	Yes	Yes	Yes	Yes	Low
Lengyel et al., 2022 [17]	Yes	Yes	Yes	No	No	Yes	Yes	Yes	Yes	Yes	High
Miclea et al., 2022 [18]	Yes	Yes	Yes	Unclear	Yes	Unclear	Yes	Yes	Yes	Yes	Moderate
Streață et al., 2022 [19]	Yes	Yes	Yes	Yes	Yes	Yes	Yes	Yes	Yes	Yes	Low
Kamath et al., 2022 [20]	Yes	Yes	Yes	Unclear	Unclear	Unclear	Yes	Unclear	Yes	Yes	High
Cucinotta et al., 2023 [21]	Yes	Yes	Yes	Yes	Yes	Yes	Yes	Yes	Yes	Yes	Low
Chaves et al., 2024 [22]	Yes	Unclear	Yes	Yes	Yes	Yes	Unclear	Yes	Yes	Yes	Moderate
Akkus and Cubuk, 2024 [23]	Yes	Unclear	Yes	Unclear	Unclear	Unclear	Unclear	Yes	Yes	Unclear	High
Lall et al., 2026 [24]	Yes	Yes	Yes	Yes	Yes	Yes	Yes	Yes	Yes	Yes	Low
Sanri et al., 2026 [25]	Yes	Yes	Yes	Yes	Yes	Yes	Yes	Yes	Yes	Yes	Low

Discussion

Principal Findings

This systematic review of 13 contemporary primary studies, encompassing 11,535 children across nine countries and three continents, found a median study-level diagnostic yield of 18.5% for pathogenic or likely pathogenic copy number variants in children investigated for unexplained developmental delay, intellectual disability or autism spectrum disorder, with individual study yields ranging from 11.0% to 37.2%. Eleven of the 13 cohorts, drawn from health systems as different as those of Brazil, South Korea, Serbia, Türkiye and India, and using commercial array platforms of differing probe density, reported yields between 14% and 23%. This is consistent with the 15-20% range projected by the 2010 international consensus statement [4], and offers some independent support for that projection in populations that differ from the predominantly North American and Western European cohorts on which the original estimate rested. It is not formal external validation: all 13 cohorts were retrospective and referral-based, four were at high risk of bias, and the confidence intervals around the smaller estimates are wide.

The second principal finding is that this headline figure conceals a phenotypic gradient steep enough that quoting a single yield to a family or a payer would be misleading. In the Chinese cohort, the yield in autism spectrum disorder was 3.3% against 31.5% in intellectual disability and developmental delay--a near tenfold difference within a single laboratory, on a single platform, applying a single classification framework [14]. The southern Brazilian cohort reproduced the gradient within the autism phenotype alone, reporting 7% for isolated ASD without intellectual disability or dysmorphism, 10% for ASD overall, and 16% where dysmorphic features accompanied the autism diagnosis [22]. The Craiova cohort reported 27.42% where global developmental delay or intellectual disability co-occurred with multiple congenital anomalies [19]. The clinical implication is that the pre-test probability of a CMA-detectable etiology is largely a function of phenotypic complexity, and pre-test counseling should be calibrated accordingly rather than delivered from a generic 15-20% figure. A related observation is that, within this evidence base, differences between array platforms appeared less influential than differences in referral phenotype: cohorts using 750K, 850K and high-density platforms returned overlapping yields, whereas cohorts differing in referral phenotype diverged by an order of magnitude. This comparison is indirect and confounded, since platform and population vary together across studies and no included cohort compared platforms head-to-head, so it should be read as a pattern worth testing rather than as a demonstration that phenotype matters more than resolution.

Yield is Not Accuracy: What This Evidence Base Cannot Support

A recurring difficulty in this literature, and one this review has tried to avoid reproducing, is the slide from statements about detection to statements about performance. Diagnostic yield answers the question "in what proportion of children referred for testing did the array return a result classified as causative?" It does not answer "how often is that classification correct?", nor "how often does a normal array correctly exclude a CNV-mediated etiology?" Answering either would require an independent reference standard, and as Table 4 records, no included study had one. The absence is structural rather than a reporting oversight: in routine practice, CMA is frequently the first and only genome-wide test a child receives, so no comparator classification of true status exists to cross-tabulate against. Where subsequent exome or genome sequencing is performed, it detects a different variant class and therefore does not function as a reference standard for CNV detection either.

The practical consequences are worth stating plainly. First, no claim in this review should be read as asserting that CMA is more accurate than any alternative; what the evidence supports is that CMA detected more abnormalities than karyotype in the one cohort where both were performed in the same children [24], with the caveats about paired analysis given in the Results. Second, the phenotypic determinants assembled in Table 5 are associations from individual cohorts, several of them univariable and none adjusted for multiple comparisons, and they are not a validated risk model. The single largest effect estimates in the review, the odds ratio of 6.13 for co-occurring cardiac defects [20], comes from 81 children in one center. Third, and looking forward, the field would benefit from exactly the analytic apparatus that is currently absent: a multivariable model combining phenotypic features into a calibrated pre-test probability of a CMA-detectable diagnosis, developed with an adequate events-per-variable ratio, validated externally rather than only internally, reported with discrimination and calibration, and appraised with a prediction-model risk-of-bias instrument. The methodological literature on high-dimensional biomedical prediction is explicit that such models fail predictably when derivation and validation data are not properly separated, when classes are imbalanced, when the feature-to-sample ratio is unfavorable, and when interpretability and calibration are neglected in favor of headline discrimination [26,27]. Any future attempt to build a pre-test probability tool for CMA referral should be designed against those standards from the outset.

Could Population Differences Explain the Variation Between Countries?

An obvious question is whether the spread of yields reflects genuine differences between populations rather than differences in ascertainment. Two observations bear on this, and both counsel caution. On the one hand, the included studies do document population-level genomic differences of the kind that could matter. The southern Brazilian cohort found long contiguous stretches of homozygosity consistent with first- to fifth-degree consanguinity in approximately 11.5% of patients [22], a background rate that differs markedly between the populations represented here and that raises the prior probability of recessive and imprinting-related diagnoses. Uniparental disomy of chromosome 15 was recurrent in both Brazilian and Romanian cohorts [15,18]. Ancestry also affects classification rather than biology alone: the population reference databases against which CNV frequency is judged remain dominated by individuals of European ancestry, so a variant common in an under-represented population may be less confidently called benign, which would tend to inflate both VUS rates and, at the margin, pathogenic calls in non-European cohorts.

On the other hand, the pattern of results does not support population genetics as the main explanation for the observed spread. The two cohorts furthest apart in yield, the Hungarian series at 37.2% and one Turkish series at 11.0% [17,23], differ most conspicuously not in ancestry but in how patients reached the test: the Hungarian cohort was assembled where CMA was not yet routine and testing was reserved for children with a high pre-test probability of a chromosomal cause, while the Turkish series counted only phenotype-associated etiological diagnoses. Conversely, the two Romanian cohorts, ascertained in the same country by different centers, agreed closely at 18.51% and 21.29% [18,19]. Ascertainment and numerator definition therefore appear to account for more of the between-study variance than population ancestry does. This remains an inference from indirect comparison; no included study was designed to separate these influences.

Comparison With Previous Literature

The pooled CMA diagnostic yield of 0.19 (95% CI 0.16-0.21) reported in a 2025 meta-analysis of 102 studies and 55,752 children with global developmental delay or intellectual disability [9] is close to the median study-level yield of 18.5% observed here, providing convergent support from an independent and much larger synthesis using different inclusion criteria. That same meta-analysis reported a pooled exome sequencing yield of 0.37, roughly double the CMA figure, and found that exome sequencing outperformed CMA in both same-sample (odds ratio 2.27) and different-sample (odds ratio 1.65) comparisons. These findings are consistent with the earlier multidisciplinary consensus statement arguing for exome sequencing as a first-tier test in neurodevelopmental disorders [7] and with the subsequent ACMG evidence-based guideline recommending ES or GS as a first- or second-tier investigation in this population [8]. On the narrow question of diagnostic yield, the weight of current evidence therefore favors sequencing over array-based testing, although the comparison rests largely on non-randomized data and the margin varies with the phenotype tested.

The practical corollary is less settled. The 13 included studies come disproportionately from settings in which sequencing is not the realistic alternative to CMA; in several, karyotype is several included cohorts explicitly framed their work as an argument for introducing CMA into routine practice in health systems where it is not yet standard: Hungary [17], Serbia [16], Romania [18,19], Brazil [15] and India [20,24]. In these contexts, the relevant comparison may not be CMA versus exome sequencing, but rather CMA versus karyotype or versus no genomic testing at all. This suggests that the first-tier question may yield different answers across economies, and that a uniform global recommendation risk limited applicability where the diagnostic deficit is largest.

Our findings on autism spectrum disorder also warrant comparison with earlier work. The 15.2% yield reported in the deeply phenotyped Italian ASD cohort [21] sits at the upper end of the 3.3-15.2% range observed across the included studies and, as those authors noted, is compatible with previously reported ASD yields spanning 9.3% to 29%. The wide historical range for ASD plausibly reflects ascertainment rather than biology: cohorts in which autism is accompanied by intellectual disability, dysmorphism, or congenital anomalies yield severalfold more than cohorts of idiopathic, non-syndromic autism. The southern Brazilian data make this explicit within one cohort [22], and it is arguably the most useful single piece of information for a clinician counseling the family of a child with isolated autism and normal cognition.

Re*l*ation to Prenatal Chromosomal Diagnosis and Imaging Phenotypes

This review was restricted to postnatal testing, and prenatal studies were excluded by protocol. That boundary is worth examining rather than simply asserting, because the prenatal literature offers an instructive contrast on the very point at issue here: the relationship between a phenotypic indication and the yield of chromosomal testing. In prenatal practice, the phenotype that triggers testing is an imaging phenotype. A recent review of ultrasonographic soft markers describes how findings such as increased nuchal translucency, absent or hypoplastic nasal bone, echogenic bowel, mild ventriculomegaly and choroid plexus cysts are used to stratify the risk of chromosomal abnormality, and how that stratification is then acted upon through conventional karyotyping, CMA, SNP array and non-invasive prenatal testing or screening [28]. Trisomy 21, trisomy 18, and structural chromosomal abnormalities were the abnormalities most often associated with soft markers on karyotyping, with trisomy 13, sex-chromosome aneuploidies, and mosaic abnormalities detected less frequently [28].

Two parallels are relevant to the present findings. First, the prenatal literature has arrived at the same structural conclusion as the postnatal cohorts synthesized here, namely that the yield of chromosomal testing is a function of the indication rather than a property of the assay: an isolated soft marker carries a substantially lower risk than multiple markers or a marker accompanied by a structural anomaly, exactly as isolated autism carries a substantially lower postnatal yield than developmental delay accompanied by dysmorphism or congenital anomalies. Second, prenatal practice has moved further than postnatal practice towards explicit multimodal risk stratification, combining an imaging phenotype with a cell-free DNA screening result and a definitive cytogenetic test in a defined sequence. The postnatal pathway for DD/ID/ASD has no comparable formalized sequence, and the phenotypic determinants cataloged in Table 5 remain a collection of single-cohort associations rather than an integrated triage algorithm. The prenatal experience suggests that such integration is achievable and that imaging and other phenotypic data can be combined with genomic testing in a structured, rather than ad hoc, manner. Because these prenatal data fall outside the eligibility criteria of this review, they are cited narratively as context and did not contribute to any quantitative statement reported here.

Interpretive Burden, Penetrance, and the Limits of Copy Number

Several findings speak to a problem that yield statistics obscure: a substantial proportion of abnormal CMA results are not diagnostically decisive. In the Cluj cohort, pathogenic findings and variants of uncertain significance together were present in 28.04% of patients, while pathogenic findings alone accounted for 18.51%, so roughly a third of abnormal results were uninterpretable at the time of reporting [18]. The southern Brazilian cohort found that 12% of children carried a rare variant of uncertain significance as their only clinically relevant finding [22], and the Serbian and New Delhi cohorts reported VUS rates of 6.7% and 2.66%, respectively [16,24]. Uncertain results are, on the available evidence, harder to communicate than either clearly positive or clearly negative ones, and the São Paulo data suggest they are also expensive to resolve: among 126 patients with an autosomal VUS in whom parental segregation was tested, 125 variants proved inherited and only a single variant was reclassified, so that 252 parental arrays produced one actionable result [15]. The authors concluded that in resource-limited settings, the marginal return on parental CMA for VUS resolution is low enough that those tests may be better spent on additional probands. That conclusion is drawn from one large cohort and would merit testing in other health systems before being adopted as policy.

The penetrance problem compounds the interpretive one. Several of the most frequently detected variants in these cohorts are susceptibility rather than deterministic alleles. In the São Paulo series, the five commonest syndromic findings included 15q13.3, 16p11.2, and 15q11.2 variants, four of the five showing incomplete penetrance and variable expressivity, and a second CNV hit was identified in 26.4% of patients carrying such a susceptibility variant [15]. This is consistent with the two-hit and oligogenic models developed from earlier large case-control work, which established that phenotypic severity in carriers of recurrent neurodevelopmental CNVs is modified by additional rare variants elsewhere in the genome [6]. A CMA result reporting a 15q11.2 deletion is therefore not equivalent, in counseling terms, to a result reporting a de novo multigenic deletion, even though both may be recorded in the same yield numerator--a heterogeneity that the ACMG/ClinGen evidence-weighted framework was designed to make explicit [5] and that the present literature reports inconsistently.

Clinical Utility: A Persistent Evidence Gap

The most striking negative finding of this review is how little the included studies say about clinical utility, despite it forming half of our review question. Diagnostic yield was the terminal reported outcome in every included cohort, and none of the 13 studies prospectively documented changes in surveillance, medication, referral, or family planning attributable to the CMA result. This gap is not inevitable, and earlier work established both the methods and the findings. It should be emphasized that the two studies cited here for this purpose were published before the review’s 2021 date limit, were not eligible for the systematic evidence synthesis, and are cited narratively as historical context only; they do not form part of the appraised evidence base. A retrospective chart review of 1,792 patients found clinically relevant results in 13.1% and quantified the proportion in which abnormal results prompted specific clinical action [29], and a Hong Kong cohort of 327 children reported that pathogenic or likely pathogenic findings prompted clinical action in 28 of 37 patients (75.7%), against only one of 40 patients with findings of uncertain significance, and proposed the diagnostic yield of clinically actionable results--8.6% in their series--as a more appropriate metric for evaluating CMA [30]. That proposal, made over a decade ago, appears not to have been widely adopted.

This pattern--an assay whose analytic capability has advanced faster than the evidence for its effect on care--is not peculiar to CMA or to pediatric neurogenetics. The same gap has been described in precision oncology, where the clinical translation of high-resolution and multi-omic tumor profiling is limited not by the technical capacity to generate the data but by the scarcity of prospective validation and by the practical difficulties of implementation [31]. The parallel is instructive because oncology has had both the funding and the trial infrastructure to close such gaps and has still found them persistent; a field with fewer resources should not expect the gap to close spontaneously. Reviews of multi-omic integration pipelines and of artificial-intelligence-enabled multimodal integration make a complementary point, emphasizing that combining data layers raises rather than lowers the evidential bar, since interpretability, generalizability and rigorous validation become harder as dimensionality increases [26,27]. CMA is a single genomic layer, and its evaluation should be settled on its own terms before it is folded into more complex integrative frameworks.

Three priorities follow from this review. Prospective cohorts should report clinically actionable yield alongside diagnostic yield, using a pre-specified action taxonomy; the clinical-report framework identifying prognostic guidance, surveillance, recurrence-risk counseling and access to condition-specific resources [3] provides a ready-made structure. Cohorts in health systems where CMA remains the practical first-tier test should routinely report phenotype-stratified yield, and economic evaluations comparing tiered strategies--CMA first with sequencing reflex versus sequencing first--are needed specifically in low- and middle-income settings, where the existing high-income cost-effectiveness literature may not transfer.

What This Means for Families and Clinicians

Stated without technical language, the findings amount to this. If a child has unexplained developmental delay, intellectual disability, or autism and is offered a chromosomal microarray, the test will find a genetic explanation in roughly one in five children overall--but the real chance depends heavily on the individual child. For a child who has autism alone, with normal learning and no unusual physical features, the chance is closer to one in fourteen. For a child who has developmental delay together with unusual facial features, a heart defect or other birth differences, it can be higher than one in four. Families deserve to be told which of these situations applies to their child before the blood test is taken, rather than a single average figure.

Families should also know that three outcomes are possible, not two. The test may find a clear cause; it may find nothing, which does not mean there is no genetic cause, only that this particular test cannot see it and that sequencing may still be informative; or it may find a change of uncertain meaning, which happens in up to about one in eight children and can be unsettling precisely because it cannot yet be interpreted. Where such a result is found, testing the parents often shows the change was inherited from a healthy parent and rarely resolves the uncertainty. Finally, and honestly, a genetic label does not by itself change day-to-day treatment for most children. Its value lies in ending years of uncertainty, in flagging specific health checks that some genetic conditions require, in giving accurate information about the chance of recurrence in a future pregnancy, and in connecting families to others with the same diagnosis. What this review could not establish, because the published studies have not measured it, is how often a microarray result actually changes what doctors do next - and that is the question the next generation of studies should be designed to answer.

Limitations

Several limitations should be considered. The evidence base was small, with only 13 studies, largely due to strict eligibility criteria and a five-year date restriction. The review was not prospectively registered in PROSPERO, although a dated internal protocol was finalized before screening, so pre-specification cannot be externally verified. All studies were retrospective and based on clinically referred cohorts, with four at high risk of bias, and referral-based ascertainment inflates yield relative to an unselected population. No study used an independent reference standard, so sensitivity, specificity, predictive values, discrimination and calibration could not be estimated; the review therefore reports detection under routine conditions rather than diagnostic accuracy, and Table 4 sets out this constraint metric by metric. Comparative performance against standard clinical testing rested on a single cohort in which both CMA and karyotype were performed in the same children, and the paired statistics needed for a formal comparison were not reported. No included study developed or validated a prediction model, so no calibrated pre-test probability estimate is available for clinical use.

In addition, the reported 18.5% yield is an unweighted median of study-level yields rather than a pooled or population-weighted estimate, and cohorts ranging from 78 to 5,778 participants contribute equally to it. CNV classification practices varied, with some cohorts including uniparental disomy or aneuploidy in the numerator and blinding of interpretation to phenotype was not reported in any study. Phenotypic determinants were largely assessed using univariable, single-cohort associations, without adjustment for multiple comparisons. The denominator for one included study was derived arithmetically by the review team rather than reported by its authors. No eligible study originated from North America or Western Europe, limiting transferability to those referral populations. Prenatal cohorts were excluded by protocol, so the findings do not speak to prenatal chromosomal diagnosis, which is discussed narratively only. One potentially eligible report was inaccessible behind a paywall, and publication bias cannot be excluded. Finally, no study directly assessed downstream clinical management, so clinical utility could only be inferred indirectly from evidence predating the review window.

## Conclusions

Across 11,535 children in nine countries, chromosomal microarray analysis returned a pathogenic or likely pathogenic copy number variant in a median of 18.5% of children investigated for unexplained developmental delay, intellectual disability, or autism spectrum disorder, with 11 of 13 cohorts falling between 14% and 23%. This consistency across markedly different health systems and array platforms supports the continued use of CMA as a practical first-line genomic test wherever sequencing is not yet accessible, while the higher yields now demonstrated for exome and genome sequencing mean that a negative microarray should not be treated as the end of the diagnostic pathway. The more actionable message is that a single average yield is the wrong number to quote. Yield falls to around 7% in isolated autism with normal cognition and rises above 27% when developmental delay is accompanied by multiple congenital anomalies. Phenotype, not the test, sets the pre-test probability, and counseling should reflect that. Three problems remain unresolved. Variants of uncertain significance complicate up to one in eight results and are expensive to resolve. No study in this literature permits estimation of sensitivity, specificity, or calibration, so the field continues to describe a test it has not formally measured, and not one of the 13 included cohorts assessed whether a microarray diagnosis altered surveillance, treatment, or reproductive counseling. Until studies report clinically actionable yield alongside diagnostic yield, and until pre-test probability is estimated by validated models rather than by single-cohort associations, the case for CMA will continue to rest on what it detects rather than on what it does for the children and families who undergo it.

## Appendices

Full electronic search strategy

All databases were searched from 1 January 2021 to 26 June 2026, which was the date of the last search. No language or publication-status filter was applied at the search stage other than the exclusion of pre-prints at screening. Concept blocks were combined with the Boolean operator AND; terms within each block were combined with OR. Truncation is indicated by an asterisk.

**Table 7:** Electronic search strategy.

Database	Interface	Search string and limits	Records retrieved
PubMed/MEDLINE	NCBI web interface	("chromosomal microarray"[tiab] OR "chromosome microarray"[tiab] OR "CMA"[tiab] OR "array CGH"[tiab] OR "array comparative genomic hybridization"[tiab] OR "SNP array"[tiab] OR "molecular karyotyping"[tiab]) AND ("developmental delay"[tiab] OR "global developmental delay"[tiab] OR "intellectual disability"[tiab] OR "mental retardation"[tiab] OR "autism"[tiab] OR "autism spectrum disorder"[tiab] OR "neurodevelopmental disorder*"[tiab]) AND ("diagnostic yield"[tiab] OR "detection rate"[tiab] OR "diagnostic rate"[tiab] OR "clinical utility"[tiab] OR "copy number variant*"[tiab] OR "CNV"[tiab]) AND ("child*"[tiab] OR "paediatric"[tiab] OR "pediatric"[tiab] OR "infant*"[tiab]) Filters: 2021/01/01–2026/06/26	202
Embase	Elsevier Embase.com	('chromosomal microarray':ti,ab OR 'chromosome microarray':ti,ab OR 'array cgh':ti,ab OR 'comparative genomic hybridization':ti,ab OR 'snp array':ti,ab OR 'molecular karyotyping':ti,ab) AND ('developmental delay':ti,ab OR 'intellectual disability':ti,ab OR 'mental deficiency':ti,ab OR 'autism':ti,ab OR 'autism spectrum disorder':ti,ab OR 'neurodevelopmental disorder*':ti,ab) AND ('diagnostic yield':ti,ab OR 'detection rate':ti,ab OR 'clinical utility':ti,ab OR 'copy number variation':ti,ab OR 'cnv':ti,ab) AND (child*:ti,ab OR paediatric:ti,ab OR pediatric:ti,ab OR infant*:ti,ab) AND (2021-2026)/py	88
Scopus	Elsevier Scopus	TITLE-ABS-KEY("chromosomal microarray" OR "chromosome microarray" OR "array CGH" OR "array comparative genomic hybridization" OR "SNP array" OR "molecular karyotyping") AND TITLE-ABS-KEY("developmental delay" OR "intellectual disability" OR "mental retardation" OR "autism" OR "autism spectrum disorder" OR "neurodevelopmental disorder*") AND TITLE-ABS-KEY("diagnostic yield" OR "detection rate" OR "diagnostic rate" OR "clinical utility" OR "copy number variant*" OR "CNV") AND TITLE-ABS-KEY(child* OR paediatric OR pediatric OR infant*) AND PUBYEAR > 2020 AND PUBYEAR < 2027	73
Web of Science	Core Collection, Clarivate	TS=("chromosomal microarray" OR "chromosome microarray" OR "array CGH" OR "array comparative genomic hybridization" OR "SNP array" OR "molecular karyotyping") AND TS=("developmental delay" OR "intellectual disability" OR "mental retardation" OR "autism" OR "autism spectrum disorder" OR "neurodevelopmental disorder*") AND TS=("diagnostic yield" OR "detection rate" OR "diagnostic rate" OR "clinical utility" OR "copy number variant*" OR "CNV") AND TS=(child* OR paediatric OR pediatric OR infant*) Timespan: 2021-01-01 to 2026-06-26	71
Total records identified	-	Duplicates removed: 267; Records screened: 167	434
